# Supplementary figures and images for: An intelligent zero trust secure framework for software defined networking
Source: PeerJ Comput Sci. 2023 Nov 17;9:e1674. doi: 10.7717/peerj-cs.1674 (PMC10703060; doi:10.7717/peerj-cs.1674)

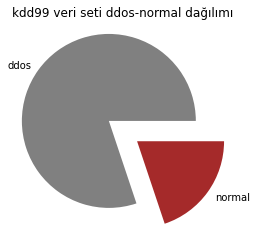

Supplement: Supplemental Information 2 [file peerj-cs-09-1674-s002.zip › Code/Detection-in-SDN-master/gorseller/kdd_veri_daglimi.png]

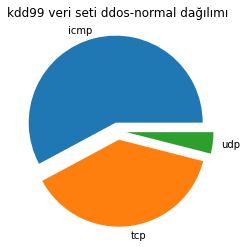

Supplement: Supplemental Information 2 [file peerj-cs-09-1674-s002.zip › Code/Detection-in-SDN-master/gorseller/protocol_type_daglimi.png]
